# Hospital Meetings, &c.

**Published:** 1897-02-13

**Authors:** 


### HOSPITAL MEETINGS, &C.

### HOSPITAL REFORM ASSOCIATION.

A meeting of the medical profession, convened by Mr. T. Garrett Horder (hon. secretary of the Hospital Reform Association), was held at St. Martin's Town Hall, W.C., on Thursday (5th inst.), to discuss the question of hospital abuse and the recommendations of the Hospital Reform Association. Dr. J. Ward Cousins (Southsea) presided.

Dr. Isambard Owen moved:—

"That the wholesale, indiscriminate, and hasty relief at present administered by many hospitals in out-patient and casualty departments is demoralising to the community, detrimental to the interests of true charity, unjust to the medical profession, and injurious to medical education; and that in the opinion of this meeting the time has arrived when the attention of governors of hospitals should be seriously given to the matter."

Dr. Owen said that with a great proportion of cases hospitals were simply doing the work which ought to be done by the Poor Law Administration, or else by the thrift of the patients themselves.

Dr. Frederick J. Smith seconded, and Mr. Jessop and Mr. Nelson Hardy supported the resolution.

In the course of the discussion which followed,

The Hon. Sydney Holland, chairman of the London Hospital, spoke in defence of the hospitals. At the London Hospital, the abuse of hospitals, of which people talked so glibly, hardly existed. He had spent day after day in the out-patient department, he had himself stopped every person who he thought was at all in a position to pay, and he was surprised and pained and grieved at the absolute misery and destitution of the people who were brought there. They knew how ready people were to stop their subscriptions and to take every possible excuse for stopping them, and he thought responsible gentlemen like those present should approach the staffs of the hospitals and not appeal to the public by meetings of that sort, held by gentlemen unconnected with London hospitals. (Cheers.)

Mr. Timothy Holmes said that at St. George's Hospital, where a good many years ago he used to see out-patients, he should say that there were very few people indeed who could pay anything like a reasonable fee.

Ultimately the resolution was carried, the words "the medical and surgical staff and" being inserted before the word "governors."

Mr. Timothy Holmes moved, and Mr. Brindley James seconded the following resolution, which was carried:—

"That, in the opinion of this meeting, the recommendations of the Hospital Reform Association for the management of the out-patient and casualty departments of our hospitals are deserving of the careful consideration of the governing bodies of the hospitals."

Dr. W. Knowsley Sibley moved, and Mr. Henry seconded the following resolution, which was carried:—

"That this meeting is of opinion that the objects of the Hospital Reform Association are worthy of the support not only of the medical profession, but of all laymen who take an interest in the welfare of our great medical charities."

The proceedings ended with a vote of thanks to the chairman.

### AFTER-CARE ASSOCIATION.

The annual meeting of the After Care Association for poor persons discharged recovered from asylums for the insane was held at 5, Hyde Park Square, n Monday, 8th inst., Archdeacon Sinclair in the chair. The council of the association reported that during 1896 they had 135 cases before them, as compared with 121 in 1895. There had been a decrease in subscriptions and donations, though there was a small balance in hand. The adoption of the report was moved by the Rev. Canon Elwyn, seconded by Dr. Nicholson, supported by the Rev. M. R. Neligan, Mr. E. Parker Young, and Dr. R. Jones, and unanimously carried. The officers for the coming year were appointed.

### CITY OF LONDON TRUSS SOCIETY.

The annual meeting of the governors of the City of London Truss Society was held on Wednesday, the 3rd inst., at the offices, 35, Finsbury Square, E.C., Mr. John Norbury, the treasurer, presiding.

From the report it would appear that the income of the institution had been fairly maintained during the past year, notwithstanding the increasing competition of younger societies. The total income of the society for the year had amounted to £4,549, and the expenditure to £6,131. The patients relieved in 1896 numbered 9,723, bringing up the total relieved since the foundation of the charity in 1807 to 520,281. The ages of patients ranged from infants of a few weeks to adults over 95. The instruments supplied were in number 9,791.

On the motion of the Chairman, seconded by Mr. G. T. Wilson, the report was adopted.

At the close of the ordinary business a special meeting was held, at which a resolution was passed empowering the committee to sell out £2,500 of the funded property of the society for the purpose of repaying loans from the bankers and to meet the general requirements of the charity.

